# Editorial: Innovations in biomaterials to reduce environmental impact

**DOI:** 10.3389/fchem.2023.1332258

**Published:** 2023-11-23

**Authors:** Thomas J. Webster, Anderson Oliveira Lobo, Afzal Hussain

**Affiliations:** ^1^ School of Health Sciences and Biomedical Engineering, Hebei University of Technology, Tianjin, China; ^2^ Division of Pre-College and Undergraduate Studies, Brown University, Boston, MA, United States; ^3^ Program in Materials Science, Federal University of Piaui, Teresina, Brazil; ^4^ Department of Pharmaceutics, King Saud University, Riyadh, Saudi Arabia

**Keywords:** biomaterials, green, environmentally friendly, global Warming, medical devices

Quiz time. What do the following materials have in common: titanium, stainless steel, polyvinyl chloride, polystyrene, polyurethane, polyethylene, polyesters, silicon, alumina, and silicone? Any guesses?

They are all biomaterials, with titanium and its alloys being the most implanted chemistry (Sterling Medical Devices, 2022).

Had trouble with that one ? Let’s try another question. What else do those chemistries above have in common ? This is a tough one also since nobody likes to talk about it, but we all know it.

They are all harming the environment. None of these materials are considered environmentally-friendly. Whether through their synthesis or use, metals, polymers, ceramics, and composites thereof leave a large carbon footprint on this Earth. And, through their manufacturing, they emit greenhouse gases or through their use, they accumulate in the so-called “Pacific plastic island”.

So putting the above two questions into one, we have one more question for you: with almost every other industry (such as automobile, construction, household products, consumer goods, aerospace, etc.) embracing green or environmentally friendly technologies and materials, to date, why hasn’t biomaterials ? This is even more puzzling considering that the biomaterials field uses some of the same materials as those originally developed for other industrial applications (such as titanium, stainless steel, polymers, etc.) and those industries have now moved away from their use since they harm the environment.

Some will say that in biomaterials our most important concern is saving human health at any means possible, using environmentally friendly or unfriendly materials. Really ? How can one save human health while in the end destroy the environment that we all count on to live ?

**FIGURE 1:**
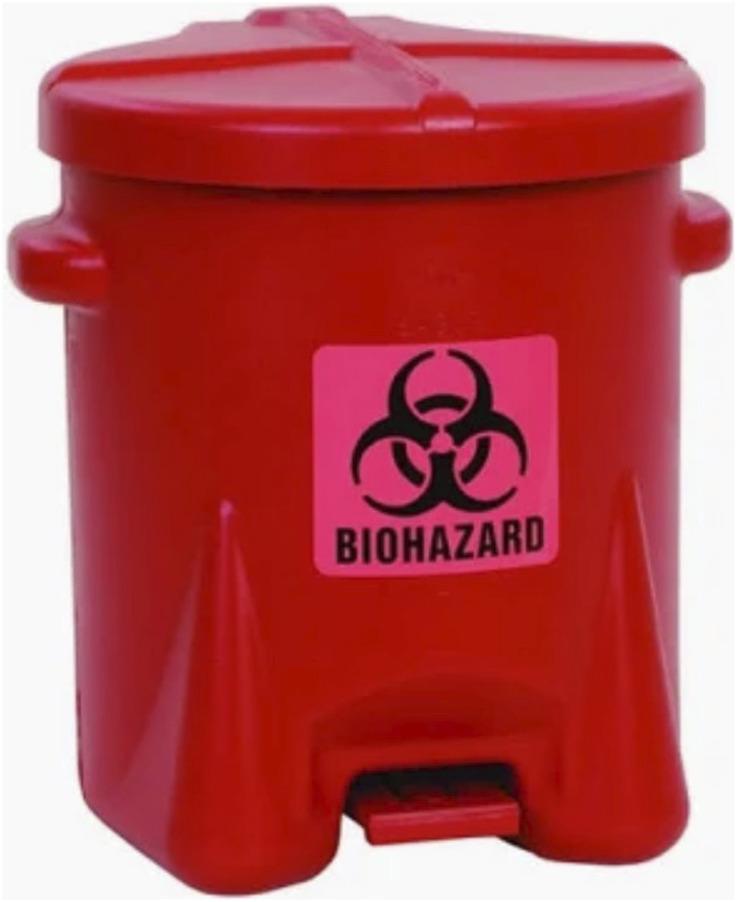
Do you know where your biohazard bags go ? Are you helping or hurting the environment in your biomaterials research ? Can you do better ?

It is clear that in terms of medical products available on the market, there has been little effort to reduce the environmental impact from biomaterials synthesized and used today. In the 1960s the first hip implant made of titanium was proposed, and guess what, today we are still implanting essentially the same titanium-based hip implant (Gomez and Morcuende, 2005). We could fill this editorial with facts showing that this must change and everyone from regulatory agencies to Universities to the medical device industry to journal publishers need to change. For example, in 2019, 850 million metric tons of greenhouse gases were produced from polymer manufacturing (many of which are used in the biomaterials field and in the development of improved biomaterials) (World Economic Forum, 2022). In 2050, this number is predicted to reach 2.8 gigatons of CO_2_ emissions (World Economic Forum, 2022). Included in this harm to the environment is the extensive use of polymers for *in vitro* testing (such as tissue culture polystyrene petri dishes). Look around your research lab. Would you call it environmentally friendly ? Is that chloroform you use to make biodegradable poly-lactic-co-glycolic acid environmentally friendly ? What happens to the biohazard bags you throw away (Figure 1)? Out of sight, out of mind ? The environment doesn’t think so. And in case you have not been paying attention, September 2023 was the highest average global temperature for the month of September since records have been kept (over 174 years) (National Oceanic and Atmospheric Administration, 2023). Do you think we have an environmental crisis ?

Don’t get us wrong. There is plenty of excellent research on green biomaterials. As true with the use of green chemistry in other fields, green biomaterials not only reduce harm to the environment, but they also perform better. Better tissue growth. Better anti-bacterial properties. Better drug delivery. Better anti-inflammation and better anti-thrombus activity, and more. However wonderful this research has been by the academic community, few if any of these attempts have been approved by medical regulatory agencies, and thus, such research is not doing anything to improve the environment.

We need a call to action. Here are some simple solutions we all can take to reverse these trends. Why don’t regulatory agencies fast track approval of green biomaterials ? Materials used for high-risk diseases are fast tracked. Isn’t destroying the Earth a risk to avoid, now ? Why don’t Universities require all *in vitro* testing be done with recycled petri dishes, 12 well plates, ELISA plates, pipettes, and more ? Universities and industries love to claim they are “green campuses”—does that include the non-green chemistries used in research and teaching labs ? Of course not. University labs are constantly reviewed by Environmental Health and Safety (EHS) Departments. Instead of closing labs for placing a sharp object in normal trash (instead of a sharps trash container), why doesn’t EHS rate and improve the “environmentally friendliness” of our labs ? Same for industry labs. Why don’t funding agencies (like NIH and NSF in the US) require a certain percentage of lab supplies adhere to environmentally friendly policies before research groups receive funding ? Why doesn’t industry adopt “environmentally friendly” labeling for medical devices like they have for consumer goods ? Why don’t clinicians demand the use of biomaterials that both save human health and the environment ? Why don’t journals only publish research from labs that adhere to and are certified for “environmentally friendly policies” ? We could go on. These are such simple steps that could make a significant positive impact on the environment, yet none of them are being done. Why ?

From using natural materials (such as plants) to cells to make nanoparticles to natural proteins to make sutures to recycled catheters, we can all do better. It is with this strong message of hope that we dedicate this Research Topic to everyone trying to save the environment through their research. We hope you enjoy reading about the successful efforts to reduce the environmental impact of biomaterials highlighted in this Research Topic. We hope you become stimulated to develop and incorporate green biomaterials into your own research wherever you are and whatever you are doing.

We hope that you too will realize that one can create environmentally friendly biomaterials without reducing biomaterial efficacy and increasing costs.

Most importantly, we hope you realize that what has been seen in other fields can be realized in biomaterials if we all focus and care just a little bit more: biomaterials created in an environmentally friendly process and with environmentally friendly materials can save the environment and help human health.
